# Cardiovascular Prognosis in Stable Patients with Cardiac Amyloidosis: A Novel and Simple Risk Score

**DOI:** 10.3390/jcm15052045

**Published:** 2026-03-07

**Authors:** Ilaria Dentamaro, Pietro Guida, Yassin Belahnech, Marco Maria Dicorato, Paolo Basile, Maria Cristina Carella, Francesco Mangini, Eduardo Urgesi, Sergio Dentamaro, Cinzia Forleo, Massimo Grimaldi, Marco Matteo Ciccone, Jose F. Rodriguez-Palomares, Andrea Igoren Guaricci

**Affiliations:** 1 University Cardiology Unit, Interdisciplinary Department of Medicine, Polyclinic University Hospital, University of Bari “Aldo Moro”, 70124 Bari, Italy; m.dicorato20@studenti.uniba.it (M.M.D.); paolo.basile@uniba.it (P.B.); e.urgesi1@studenti.uniba.it (E.U.); cinzia.forleo@uniba.it (C.F.); marcomatteo.ciccone@uniba.it (M.M.C.); andreaigoren.guaricci@uniba.it (A.I.G.); 2 Cardiology Division, Miulli Hospital, 70021 Acquaviva delle Fonti, Italy; p.guida@miulli.it (P.G.); francescomangini.78@libero.it (F.M.); m.grimaldi@miulli.it (M.G.); 3 Departamento de Cardiología, Hospital Universitari Vall d’Hebron, 08035 Barcelona, Spain; yassin.belahnech@gmail.com (Y.B.); jfrodriguezpalomares@gmail.com (J.F.R.-P.); 4 Vall Hebrón Institut de Recerca (VHIR), Universitat Autònoma de Barcelona, 08193 Bellaterra, Barcelona, Spain; 5 Vascular Surgery, Polyclinic University Hospital, University of Bari “Aldo Moro”, 70124 Bari, Italy; sergio.dentamaro@gmail.com

**Keywords:** cardiac amyloidosis, heart failure hospitalization, risk stratification, electrocardiography, echocardiography, prognostic score, outpatients

## Abstract

**Background:** Cardiac amyloidosis (CA) is frequently diagnosed in clinically stable patients, yet the risk of subsequent heart failure (HF) hospitalization remains difficult to predict using readily available tools. Early identification of high-risk outpatients is crucial to optimize follow-up and therapeutic strategies. **Purpose:** To develop a simple, non-invasive risk score to predict HF hospitalization in stable patients with cardiac amyloidosis using standard electrocardiographic and echocardiographic parameters. **Methods:** We prospectively enrolled 100 consecutive patients with confirmed cardiac amyloidosis from three tertiary centers. Baseline evaluation included clinical assessment, electrocardiography, and transthoracic echocardiography. The primary endpoint was HF hospitalization during follow-up; secondary endpoints were HF-related and all-cause mortality. Cox regression analysis was used to identify independent predictors of HF hospitalization and to derive a point-based risk (CAMY-HF) score. **Results:** During a median follow-up of 36 months, 55% of patients required HF hospitalization and 47% died. Low QRS voltage, interventricular septal thickness ≥14 mm, and left ventricular ejection fraction ≤40% independently predicted HF hospitalization and were incorporated into the CAMY-HF score (range, 0–4). HF hospitalization occurred in 0% of low-risk, 47.9% of intermediate-risk, and 80.6% of high-risk patients at 3 years. Higher CAMY-HF scores were also associated with increased HF-related and all-cause mortality. **Conclusions:** The CAMY-HF score is a simple, widely applicable tool for early risk stratification in clinically stable patients with cardiac amyloidosis. By relying on routine ECG and echocardiographic parameters, it may help identify outpatients at high risk of HF hospitalization and guide follow-up intensity and management strategies.

## 1. Introduction

Cardiac amyloidosis (CA) is a progressive infiltrative cardiomyopathy caused by extracellular deposition of amyloid fibrils within the myocardium. Cardiac impairment represents a major determinant of prognosis in systemic amyloidosis [1,2]. Despite the growing awareness and advancements in diagnostic protocols, heart involvement continues to be associated with a significant degree of morbidity and mortality, primarily driven by progressive heart failure (HF) [3,4]. Hospitalization for HF constitutes a pivotal juncture in the clinical progression of CA, denoting the progression of the illness and substantially elevating the risk of mortality [5]. A considerable number of patients diagnosed with CA are found to be clinically stable or only mildly symptomatic at the time of diagnosis. However, a significant proportion of these patients will subsequently experience HF decompensation within a relatively short time frame [6]. Consequently, the identification of patients at the highest risk among those who appear to be stable outpatients remains a significant unmet clinical necessity. Over the past two decades, several prognostic staging systems have been developed for patients with cardiac amyloidosis, particularly in AL and ATTR subtypes. In AL amyloidosis, the Mayo Clinic staging system—based on circulating biomarkers such as NT-proBNP and cardiac troponins—has become the reference model for risk stratification, primarily predicting overall survival and early mortality [7]. Similarly, in transthyretin amyloidosis, staging systems incorporating NT-proBNP and estimated glomerular filtration rate have been proposed to refine prognostic assessment [8]. These biomarker-based models provide robust and reproducible prognostic information and are widely adopted in specialized centers. However, they are predominantly focused on mortality prediction and do not specifically address the risk of heart failure hospitalization, which represents a pivotal and clinically actionable event in the natural history of CA. In parallel, advanced imaging techniques have demonstrated incremental prognostic value. In both AL and ATTR amyloidosis, myocardial deformation parameters like global longitudinal strain and cardiac magnetic resonance-derived indices of tissue characterization have been linked to poor outcomes [9,10,11]. Furthermore, ventricular–pulmonary arterial coupling and right ventricular function have become highly significant indicators of disease progression and mortality [12,13]. Although these modalities greatly improve pathophysiological comprehension and prognostic precision, their accessibility may be limited by the necessity of specialized software, technical expertise, and optimal image quality. Additionally, not all outpatient workflows incorporate them, particularly in non-tertiary care settings. Consequently, there is a pressing need for a risk stratification tool that is both simple and pragmatic, and which is applicable to a wide range of cases. This tool must be specifically designed to predict hospitalization for heart failure in stable patients with cardiac amyloidosis. The development of such a tool is predicated on the utilization of universally available parameters during routine cardiology evaluations, the reproducibility of these parameters across centers, and the capacity for facile interpretation without the necessity of advanced imaging platforms. A preliminary diagnostic instrument based on electrocardiogram (ECG) and echocardiography findings could function as an initial triage instrument. This instrument would serve to complement, rather than replace, established biomarker-based staging systems and advanced imaging techniques [14]. The aim of this study was to identify clinically stable CA patients with higher risk of HF hospitalization during intermediate-term follow-up. To this end, a straightforward and practical risk stratification tool was developed. The Cardiac Amyloidosis Heart Failure (CAMY-HF) score was created based exclusively on routinely available electrocardiographic and echocardiographic parameters. The investigation further encompassed the capacity of the CAMY-HF score to predict both HF-related and all-cause mortality.

## 2. Materials and Methods

We prospectively enrolled 100 consecutive adult patients (age >18 years) with a confirmed diagnosis of cardiac amyloidosis, referred from cardiology outpatient clinics to echocardiography laboratories between 2018 and 2021 across three tertiary care centers. The diagnosis of amyloidosis was established based on histological and/or genetic confirmation. Specifically, AL amyloidosis was diagnosed in the presence of plasma cell dyscrasia—defined by detection of monoclonal protein on serum and/or urine immunofixation and/or abnormal serum free light chain ratio—together with histological confirmation of amyloid deposition and exclusion of transthyretin (TTR) gene mutations. Hereditary transthyretin amyloidosis (ATTRm) was defined by identification of a pathogenic TTR gene mutation. Wild-type transthyretin amyloidosis (ATTRwt) was diagnosed in the absence of plasma cell dyscrasia and TTR mutations, in the presence of cardiac amyloid involvement and positive 99mTc-DPD scintigraphy. Patients with alternative causes of left ventricular hypertrophy—including hypertension, aortic stenosis, hypertrophic cardiomyopathy, athletic heart, or other cardiac infiltrative diseases—were excluded. Written informed consent was obtained from all participants at the time of enrollment. The study was conducted in accordance with the principles outlined in the Declaration of Helsinki and was approved by the institutional review board of each participating center. At the time of patient enrollment (2018–2021), disease-modifying therapies for transthyretin amyloidosis were not yet widely available in routine clinical practice within the participating centers. Therefore, patients with ATTR amyloidosis were primarily managed with supportive heart failure therapy according to standard care. Similarly, patients with AL amyloidosis received conventional hematological management as indicated, but contemporary targeted therapies were not uniformly implemented. As a result, the cohort largely reflects the natural clinical course of cardiac amyloidosis under standard supportive management. Baseline clinical evaluation included demographic characteristics, cardiovascular risk factors, medical history, and New York Heart Association (NYHA) functional class. A comprehensive laboratory workup was performed at baseline, including high-sensitivity cardiac troponin T, N-terminal pro–B-type natriuretic peptide (NT-proBNP), serum creatinine, and estimated glomerular filtration rate calculated using the MDRD formula. In patients with suspected or confirmed AL amyloidosis, serum and urine immunofixation and serum free light chain measurements were systematically obtained. Standard 12-lead electrocardiography was performed in all patients. ECG parameters of interest included QRS voltage, pseudo-infarction patterns, and conduction abnormalities. Low QRS voltage was defined as QRS amplitude <5 mm in all limb leads and/or <10 mm in all precordial leads, according to established criteria [15]. Pseudo-infarction pattern was defined as the presence of pathological Q waves in the absence of a documented history of myocardial infarction. All patients underwent comprehensive transthoracic echocardiography using commercially available ultrasound systems. Image acquisition and measurements were performed according to current American Society of Echocardiography and European Association of Cardiovascular Imaging recommendations [14]. Left ventricular dimensions, wall thickness, and ejection fraction were assessed using standard methods. Left ventricular ejection fraction was calculated using the biplane Simpson method. Measurements were averaged over three cardiac cycles in sinus rhythm and five cycles in atrial fibrillation. Interventricular septal thickness was measured at end-diastole in the parasternal long-axis view. Diastolic function was evaluated using transmitral Doppler parameters and categorized as normal, impaired relaxation, pseudo-normal, or restrictive filling patterns. Global longitudinal strain (GLS) was assessed using vendor-independent software and was feasible in 98% of the study population. However, GLS was not pre-specified as a mandatory variable for score development, as the primary aim was to construct a risk model based on universally available and immediately interpretable ECG and standard echocardiographic parameters. Patients were followed at 6, 12, 24, and 36 months through outpatient visits and/or structured telephone interviews. Outcome data were verified through review of hospital records and, when necessary, direct contact with patients or their relatives to ensure completeness and accuracy of event ascertainment. The primary endpoint was hospitalization for heart failure decompensation. Secondary endpoints included heart failure–related mortality and all-cause mortality. Heart failure hospitalization was defined as admission for worsening HF symptoms requiring intravenous therapy, supported by objective clinical findings and/or radiological or laboratory evidence of congestion, as adjudicated by the treating physicians.

## 3. Statistical Analysis

Continuous variables are presented as mean ± standard deviation. Group comparisons for continuous variables were performed using parametric or non-parametric tests, as appropriate based on data distribution. Associations between categorical variables were assessed using the chi-squared test or Fisher’s exact test, as applicable. Time-to-event analyses were conducted using Cox proportional hazards regression models, as commonly applied in prognostic modeling for heart failure populations [15,16]. The proportional hazards assumption was verified using Schoenfeld residuals. Variables significantly associated with HF hospitalization at univariate analysis were entered into multivariate models. To reduce the risk of model overfitting given the sample size and number of events, the number of variables included in the final multivariate model was deliberately limited to those showing the strongest and most consistent associations at univariate analysis. Kaplan–Meier survival curves were generated and compared using the log-rank test. Model discrimination was assessed using Harrell’s C-index for time-to-event data. Formal calibration analyses were not performed given the exploratory nature and limited sample size of the study. Therefore, external validation is required before clinical implementation. To develop the risk stratification algorithm, a point-based scoring system was constructed by assigning integer values to variables that independently predicted heart failure hospitalization during follow-up. These point assignments were derived from the β-coefficients of the final multivariate Cox model and subsequently rounded to the nearest integer to ensure clinical usability. A two-sided *p*-value < 0.05 was considered statistically significant. Interventricular septal thickness was initially analyzed as a continuous variable in the Cox proportional hazards models. For the purpose of risk score construction and to enhance clinical applicability, the variable was subsequently dichotomized. The ≥14 mm cut-off was selected based on the observed risk gradient across septal thickness values in the study cohort and its consistency with established echocardiographic thresholds indicating abnormal myocardial thickening in infiltrative cardiomyopathies. This approach allowed transformation of a continuous predictor into a clinically usable categorical variable for score implementation. All statistical analyses were performed using STATA software, version 16 (StataCorp LLC, College Station, TX, USA).

## 4. Results

### 4.1. Baseline Clinical, Electrocardiographic, and Echocardiographic Characteristics

The study population consisted of 100 patients with confirmed cardiac amyloidosis, including 31% with AL amyloidosis, 36% with wild-type transthyretin amyloidosis (ATTRwt), and 33% with hereditary transthyretin amyloidosis (ATTRm). Baseline clinical, electrocardiographic, and echocardiographic characteristics are summarized in Table 1. Mean age was 68 ± 18 years, and 75% of patients were male. At baseline, most patients were clinically stable, with 75% classified as NYHA functional class I–II. Global longitudinal strain analysis was feasible in 98% of patients. During a median follow-up of 36 months (95% CI, 18–54), 55% of patients required hospitalization for heart failure, and 47% died. Both heart failure hospitalization and mortality rates were highest among patients with AL and ATTRwt amyloidosis, whereas patients with ATTRm showed a more favorable clinical course. Heart failure hospitalization occurred in more than half of the study population and represented the most frequent adverse event during follow-up. Results of the univariate Cox regression analysis are summarized in Table 2, while the full set of tested variables is provided in Supplementary Table S1. At univariate analysis, age, low QRS voltage, pseudo-myocardial infarction pattern, interventricular septal thickness, posterior wall thickness, reduced left ventricular ejection fraction, and pseudonormal diastolic filling pattern were significantly associated with heart failure hospitalization. In contrast, global longitudinal strain and several other clinical variables did not show a significant association.

**Figure 1:**
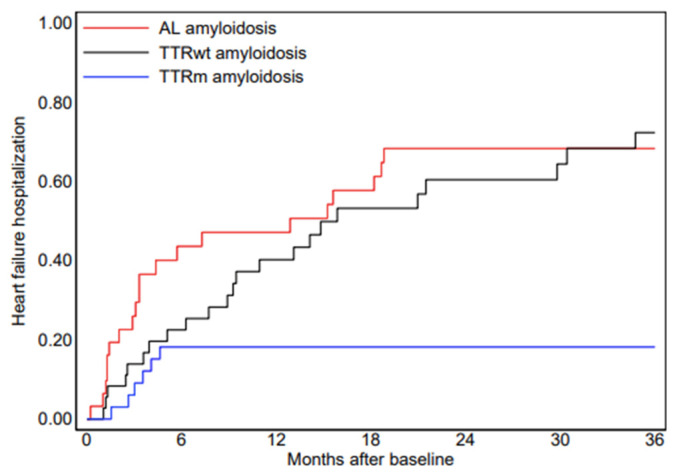
Cumulative event curve for heart failure hospitalization in the AL, ATTRwt and ATTRm group at 36 months of follow-up. The comparison between curves was statistically significant (*p* < 0.001).

At multivariate analysis, only three variables remained independently associated with HF hospitalization: low QRS voltage (HR, 3.50; 95% CI, 1.92–6.38; *p* < 0.001), interventricular septal thickness (HR, 1.12 per mm; 95% CI, 1.05–1.19; *p* < 0.001), and left ventricular ejection fraction (HR, 0.96 per %; 95% CI, 0.94–0.99; *p* = 0.003). The cumulative incidence of HF hospitalization differed significantly among amyloidosis subtypes, with higher rates observed in AL and ATTRwt amyloidosis compared with ATTRm amyloidosis (*p* < 0.001) (Figure 1). Amyloid subtype was associated with different event rates, likely reflecting differences in age distribution and disease phenotype across AL, ATTRwt, and ATTRm forms. However, subtype was not included in the final multivariate model, as the objective of the CAMY-HF score was to capture cardiac structural and functional severity irrespective of etiological classification. Moreover, given the limited sample size and number of events, inclusion of subtype as a categorical variable would have increased model complexity and the risk of overfitting. In a sensitivity analysis including amyloid subtype in the multivariate model (with ATTRm as reference), low QRS voltage, interventricular septal thickness ≥ 14 mm, and LVEF ≤ 40% remained independently associated with HF hospitalization (Supplementary Table S2), supporting the robustness of CAMY-HF beyond etiological classification.

### 4.2. Development of the CAMY-HF Score

**Figure 2:**
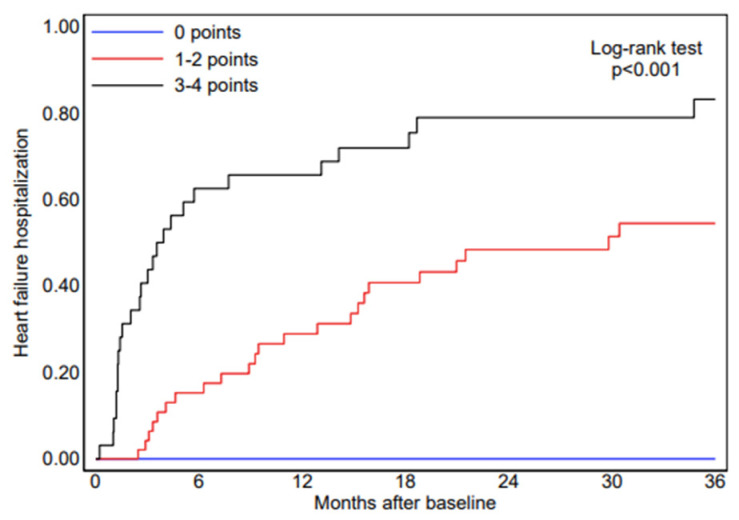
Kaplan–Meier analysis for the probability of future heart failure hospitalization according to CAMY-HF score categories. Patients were divided into low-, intermediate-, and high-risk groups. The log-rank test showed a statistically significant difference among groups (*p* < 0.001).

**Figure 3:**
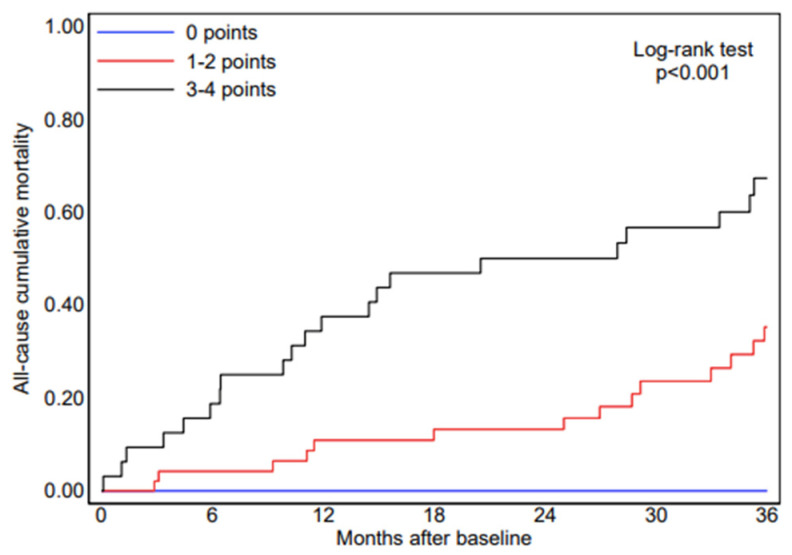
Kaplan–Meier analysis for the probability of all-cause death according to CAMY-HF score categories. A stepwise increase in mortality was observed across risk groups. The log-rank test demonstrated a statistically significant difference among groups (*p* < 0.001).

**Figure 4:**
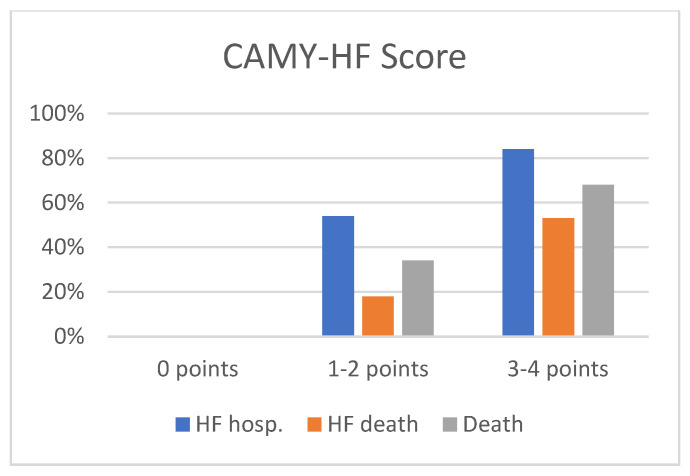
Incidence of adverse events (HF hospitalization, HF-related death, and all-cause death) across CAMY-HF risk groups. Differences among groups were statistically significant (*p* < 0.001).

Based on the independent predictors identified in the multivariate Cox regression model (Table 3), a point-based risk stratification tool—the CAMY-HF score—was developed. For score construction, interventricular septal thickness was re-entered into the Cox model as a dichotomous variable (≥14 mm). In this model specification, septal thickness ≥14 mm was associated with a hazard ratio of 9.28 for HF hospitalization, which formed the basis for point allocation. The score incorporates three routinely available parameters: low QRS voltage on electrocardiography, interventricular septal thickness ≥14 mm, and left ventricular ejection fraction ≤ 40%, resulting in a total score ranging from 0 to 4 points (Table 4). The selected LVEF threshold is consistent with established prognostic stratification in heart failure populations [17]. Patients with high CAMY-HF scores (3–4 points) had a markedly higher risk of HF hospitalization compared with those with low or intermediate scores (1–2 points). Specifically, HF hospitalization occurred in 0 of 21 patients (0%) in the low-risk group (score of 0), 23 of 48 patients (47.9%) in the intermediate-risk group (score of 1–2), and 25 of 31 patients (80.6%) in the high-risk group (score of 3–4) (Figure 2). Discrimination of the CAMY-HF score for HF hospitalization was assessed using Harrell’s C-index, which was 0.76, indicating good prognostic discrimination. Higher CAMY-HF scores were also associated with worse secondary outcomes. Kaplan–Meier survival analysis demonstrated a stepwise increase in heart failure–related mortality and all-cause mortality across increasing CAMY-HF score categories (Figure 3 and Figure 4), consistent with previous evidence supporting the prognostic relevance of ECG and echocardiographic markers in cardiac amyloidosis [12,15]. At three years of follow-up, patients in the high-risk CAMY-HF group showed substantially higher rates of HF hospitalization, HF-related death, and all-cause death compared with patients in the low- and intermediate-risk groups (Figure 4).

## 5. Discussion

In this prospective multicenter study, we developed a simple and pragmatic risk score to predict heart failure (HF) hospitalization in clinically stable patients with cardiac amyloidosis. The main finding of the study is that a restricted array of routinely obtainable electrocardiographic and echocardiographic parameters—namely, low QRS voltage, augmented interventricular septal thickness, and diminished left ventricular ejection fraction—permits the early identification of patients at elevated risk of HF decompensation during intermediate-term follow-up. Notably, the CAMY-HF score was designed to reflect cardiac structural and functional impairment irrespective of amyloid subtype, thus focusing on phenotypic cardiac severity rather than etiological classification. Crucially, the CAMY-HF score focuses on stable outpatients, a group for whom prompt risk assessment is challenging yet clinically essential, particularly in the era of disease-modifying treatments. Hospitalization for HF constitutes a critical event in the natural history of cardiac amyloidosis, signifying the advancement of the disease and a substantial increase in the risk of mortality in the future. Regardless of the specific type of amyloidosis, prior research has demonstrated that patients with cardiac amyloidosis often experience rapid clinical deterioration following their initial hospitalization for HF [2,3,4]. Notably, an increasing number of patients are diagnosed at an early or mildly symptomatic stage. However, current clinical tools lack the capacity to reliably identify which of these stable individuals will soon develop overt HF. The findings of this study underscore the necessity for the prompt implementation of risk stratification strategies for HF-related events in an outpatient setting. Furthermore, these strategies should not be limited to mortality as a sole metric. A multitude of prognostic models have been advanced for risk stratification in CA. Biomarker-based staging systems incorporating NT-proBNP and troponin levels have gained widespread acceptance due to their ability to provide reliable prognostic information [16]. In a similar vein, sophisticated imaging methods such as cardiac magnetic resonance and myocardial strain analysis have shown incremental value in predicting unfavorable outcomes [13,18]. Nonetheless, these methodologies are often influenced by extracardiac factors or center-specific expertise, and their accessibility during the initial outpatient evaluation may not be guaranteed. In addition, recent research has emphasized the prognostic significance of ventricular-pulmonary coupling abnormalities and heart failure-related events in cardiac amyloidosis, highlighting the necessity for practical risk stratification tools that can be utilized in the outpatient setting [19]. By offering a quick, first-line risk assessment based on widely available ECG and echocardiographic parameters, the CAMY-HF score is meant to supplement current staging systems rather than to replace them. Each component of the CAMY-HF score reflects a key pathophysiological aspect of cardiac amyloidosis. Increased interventricular septal thickness reflects amyloid burden and myocardial stiffening, two major determinants of diastolic dysfunction and progressive heart failure. The selection of a dichotomous septal thickness threshold was intended to enhance practical usability of the score rather than to redefine diagnostic criteria. A well-known indicator of myocardial amyloid infiltration, low QRS voltage has been repeatedly linked to poor outcomes in all forms of amyloidosis [12,15]. A subset of patients with more advanced myocardial involvement and impaired systolic reserve are identified by reduced left ventricular ejection fraction, which is frequently preserved in early disease but carries a significantly higher risk of hospitalization for heart failure [17]. Growing evidence supports the prognostic value of advanced echocardiographic and cardiac magnetic resonance parameters, including global longitudinal strain, right ventricular function, and myocardial tissue characterization [20,21]. In particular, right ventricular–pulmonary arterial coupling and deformation indices have emerged as powerful predictors of mortality in cardiac amyloidosis. In the present study, although GLS was feasible in the vast majority of patients, it was not incorporated into the CAMY-HF score. This choice was made to preserve model parsimony and to develop a tool based on universally interpretable parameters in routine outpatient practice. Future studies should explore whether integration of GLS into simplified risk models may further refine prognostic stratification, particularly in the era of contemporary disease-modifying therapies and earlier-stage diagnosis of cardiac amyloidosis. The strength of the CAMY-HF score lies in its simplicity and reproducibility, allowing early triage of patients who may subsequently benefit from more advanced imaging or closer surveillance. Notably, the score was derived in a cohort largely untreated with modern disease-modifying therapies, thereby reflecting intrinsic disease severity rather than treatment response. The identification of high-risk individuals may result in the implementation of enhanced monitoring procedures, the optimization of HF therapy prior to its onset, the timely referral of patients to specialized medical centers, or the consideration of disease-modifying treatments prior to the occurrence of overt HF decompensation. Conversely, patients with low CAMY-HF scores may be safely monitored with standard surveillance strategies.

## 6. Limitations

Several limitations should be acknowledged. First, although the number of heart failure events allowed multivariable modeling with an acceptable events-per-variable ratio, the overall sample size was relatively limited, which may increase the risk of overfitting and reduce generalizability. For this reason, the number of predictors included in the final model was deliberately restricted to the most robust variables identified at univariate analysis. Second, no formal internal validation procedures were performed, and model calibration was not formally assessed. Although discrimination was evaluated, the performance and stability of the CAMY-HF score require confirmation in larger, independent cohorts before clinical implementation. Third, the study population was derived from tertiary referral centers, potentially introducing selection bias. Although amyloid subtype influenced event rates, the study was not powered for subtype-stratified multivariable analyses, and collinearity with age and structural cardiac parameters cannot be excluded. Accordingly, the incremental prognostic value of the CAMY-HF score beyond etiological classification warrants further investigation. The present findings should therefore be considered hypothesis-generating.

## 7. Conclusions

The CAMY-HF score is a simple, non-invasive, and widely applicable tool for early risk stratification in clinically stable patients with cardiac amyloidosis. Relying exclusively on standard ECG and echocardiographic parameters, it enables identification of patients at increased risk of HF hospitalization and adverse outcomes, potentially guiding follow-up strategies and therapeutic decision-making in routine clinical practice.

## Figures and Tables

**Table 1:** Baseline clinical, electrocardiographic and echocardiographic characteristics of the study population.

Variable	Overall (*n* = 100)	AL (*n* = 31)	ATTRwt (*n* = 36)	ATTRm (*n* = 33)
Age, years	68 ± 18	70 ± 11	81 ± 5	54 ± 17
Female sex	25 (25%)	11 (35.5%)	5 (13.9%)	9 (26.5%)
NYHA class I–II	75 (75%)	23 (74%)	27 (75%)	25 (76%)
Atrial fibrillation	28 (28%)	6 (19%)	19 (53%)	3 (9%)
Hypertension	57 (57%)	20 (65%)	28 (78%)	9 (27%)
Diabetes mellitus	18 (18%)	9 (29%)	7 (19%)	2 (6%)
Low QRS voltage	26 (27%)	14 (45%)	8 (22%)	4 (13%)
Pseudo-infarction pattern	41 (42%)	15 (48%)	17 (47%)	9 (30%)
Interventricular septum, mm	16.1 ± 4.5	15.4 ± 2.0	19.2 ± 3.5	13.4 ± 5.0
LVEF, %	52 ± 12	51 ± 13	50 ± 12	55 ± 13
Diastolic dysfunction (≥pseudo-normal)	46 (46%)	13 (42%)	18 (50%)	15 (45%)
Pericardial effusion (any)	14 (14%)	8 (28%)	2 (6%)	4 (12%)

Data are baseline values and are presented as mean ± SD or *n* (%). NYHA: New York Heart Association; LVEF: left ventricular ejection fraction.

**Table 2:** Univariate Cox regression analysis for heart failure hospitalization (N = 100).

	HR (95%CI)	*p*
Female	1.31 (0.67–2.56)	0.434
Age (years)	1.03 (1.01–1.05)	**0.008**
AL amyloidosis	1.68 (0.94–2.98)	0.078
TTRwt amyloidosis	1.02 (0.52–2.01)	0.950
TTRm amyloidosis	0.26 (0.09–0.77)	**0.015**
Atrial fibrillation	1.74 (0.94–3.21)	0.078
Low voltage	3.26 (1.79–5.95)	**<0.001**
Pseudo-Myocardial Infarction	2.36 (1.30–4.27)	**0.005**
High-Voltage QRS	0.79 (0.43–1.46)	0.457
Interventricular septum (mm)	1.11 (1.04–1.18)	**0.001**
Posterior wall (mm)	1.13 (1.05–1.21)	**0.001**
Left ventricular end-diastolic volume (mL)	0.99 (0.99–1.00)	0.156
Left ventricular end-systolic volume (mL)	0.99 (0.98–1.00)	0.232
Left ventricular ejection fraction (%)	0.97 (0.94–0.99)	**0.007**
Global Longitudinal Strain	0.99 (0.92–1.08)	0.898

Analysis of heart failure hospitalization during the first 3 years of follow-up using univariate Cox proportional hazards regression. Data are presented as number and percentage or mean ± standard deviation. A *p* value < 0.05 was considered statistically significant.

**Table 3:** Multivariate Cox regression analysis for HF hospitalization.

Variable	Hazard Ratio (95% CI)	*p* Value
Low QRS voltage	3.50 (1.92–6.38)	<0.001
Interventricular septum (per mm)	1.12 (1.05–1.19)	<0.001
Left ventricular ejection fraction (per %)	0.96 (0.94–0.99)	0.003

Multivariate Cox proportional hazards model for heart failure hospitalization during follow-up. CI: confidence interval.

**Table 4:** CAMY-HF risk score for prediction of HF hospitalization.

Parameter	Hazard Ratio	Points
Low QRS voltage	3.67	1
Interventricular septum ≥ 14 mm	9.28	2
LVEF ≤ 40%	3.30	1
Total score range		0–4
**CAMY-HF score**	**Estimated 3-year HF hospitalization risk**	
0	0%	
1–2	47.9%	
3–4	80.6%	

LVEF assessed by biplane Simpson method.

## Data Availability

The data presented in this study are available on reasonable request from the corresponding author. The data are not publicly available due to privacy and ethical restrictions.

## Supplementary Materials

The following supporting information can be downloaded at: https://www.mdpi.com/article/10.3390/jcm15052045/s1. Table S1: Full univariate Cox regression analysis for heart failure hospitalization (N = 100). Table S2: Sensitivity multivariate Cox analysis including amyloid subtype.

## Abbreviations

The following abbreviations are used in this manuscript:

AL	Light-chain amyloidosis
ATTR	Transthyretin amyloidosis
ATTRm	Hereditary transthyretin amyloidosis
ATTRwt	Wild-type transthyretin amyloidosis
CA	Cardiac amyloidosis
CAMY-HF	Cardiac Amyloidosis Heart Failure
CI	Confidence interval
ECG	Electrocardiography
HF	Heart failure
HR	Hazard ratio
LV	Left ventricle
LVEF	Left ventricular ejection fraction
NYHA	New York Heart Association
