# Supplementary material for: Cardiovascular Prognosis in Stable Patients with Cardiac Amyloidosis: A Novel and Simple Risk Score
Source: J Clin Med. 2026 Mar 7;15(5):2045. doi: 10.3390/jcm15052045 (PMC12986365; doi:10.3390/jcm15052045)
Supplement: Supplementary file 1 [file jcm-15-02045-s001.zip › jcm-4164518-supplementary.pdf]

Journal of
Clinical Medicine

MDPI

Supplementary Materials:

Supplementary Table S1: Full univariate Cox regression analysis for heart failure hospitalization (N = 100).

	**n=51**	**n=49**	**HR (95%CI)**	**p**
Female	25.5%	24.5%	1.31 (0.67-2.56)	0.434
Age (years)	62±21	74±11	1.03 (1.01-1.05)	**0.008**
AL amyloidosis	21.6%	40.8%	1.68 (0.94-2.98)	0.078
TTRwt amyloidosis	25.5%	46.9%	1.02 (0.52-2.01)	0.950
TTRm amyloidosis	52.9%	12.2%	0.26 (0.09-0.77)	**0.015**
Body Surface Area (m2)	1.79±.19	1.76±.21	0.63 (0.11-3.50)	0.596
Body Mass Index (Kg/m2)	25.6±3.7	26.2±4.9	0.98 (0.90-1.06)	0.653
Obesity	12.5%	19.1%	0.99 (0.41-2.37)	0.975
NYHA I	43.1%	10.2%	0.39 (0.14-1.10)	0.076
NYHA II	43.1%	57.1%	2.57 (0.91-7.28)	0.076
NYHA III-IV	13.7%	32.7%	1.38 (0.72-2.65)	0.326
Smoker	31.4%	40.8%	1.63 (0.87-3.06)	0.124
Hypertension	47.1%	67.3%	0.76 (0.36-1.57)	0.455
Diabetes Mellitus	15.7%	20.4%	0.94 (0.45-1.99)	0.873
Dyslipidaemia	35.3%	44.9%	1.24 (0.70-2.21)	0.465
Ischemic cardiopathy	5.9%	18.4%	1.16 (0.52-2.59)	0.716
Previou sstroke	9.8%	16.3%	1.08 (0.49-2.36)	0.850
Vascular disease	5.9%	2.0%	0.27 (0.04-1.96)	0.193
Atrial fibrillation	11.8%	44.9%	1.74 (0.94-3.21)	0.078
Pacemaker	2.0%	2.0%	0.35 (0.04-2.80)	0.325
Low voltage	13.7%	38.8%	3.26 (1.79-5.95)	**0.000**
Pseudo-Myocardial Infarction	27.5%	55.1%	2.36 (1.30-4.27)	**0.005**
High-Voltage QRS	28.0%	41.7%	0.79 (0.43-1.46)	0.457
Left bundle branch block	5.9%	16.3%	0.98 (0.42-2.26)	0.956
Right bundle branch block	22.0%	24.5%	0.78 (0.40-1.54)	0.473
Left ventricular end-diastolic diameter (mm)	46±8	45±8	0.96 (0.92-1.01)	0.088
Left ventricular end-systolic diameter (mm)	30±9	33±9	0.99 (0.94-1.04)	0.638
Interventricular septum (mm)	14.1±4	18.2±4	1.11 (1.04-1.18)	**0.001**
Posteriorwall (mm)	12.4±3.7	16.4±3.3	1.13 (1.05-1.21)	**0.001**
Left ventricular end-diastolic volume (ml)	97±60	91±44	0.99 (0.99-1.00)	0.156
Left ventricular end-systolic volume (ml)	44±46	50±35	0.99 (0.98-1.00)	0.232
Left ventricular ejection fraction (%)	57±11	47±12	0.97 (0.94-0.99)	**0.007**
Normal EA	52.5%	12.8%	0.28 (0.11-0.73)	**0.009**

Reversed EA	22.5%	4.3%	0.25 (0.06-1.05)	0.058
Pseudonormal EA	25.0%	83.0%	6.01 (2.52-14.32)	**<0.001**
E/A ratio	1.3±.9	2.2±1	1.28 (0.90-1.84)	0.174
No pericardial effusion	90.0%	81.3%	0.53 (0.25-1.14)	0.104
Mild pericardial effusion	8.0%	12.5%	1.60 (0.67-3.84)	0.290
Moderate-severe pericardial effusion	2.0%	6.3%	2.26 (0.64-7.97)	0.204
Left Atrial Size (mm)	24±6	29±7	1.05 (1.00-1.09)	0.056
Global Longitudinal Strain	-14.2±4.9	-11.9±4	0.99 (0.92-1.08)	0.898

Univariate Cox proportional hazards regression analysis including all tested clinical, electrocardiographic, and echocardiographic variables. Data are presented as hazard ratio (HR) with 95% confidence interval (CI). A two-sided p-value < 0.05 was considered statistically significant.

Supplementary Table S2: Sensitivity multivariate Cox analysis including amyloid subtype

Variable	HR (95% CI)	p value
Low voltage	2.99 (1.60–5.59)	0.0006
IVS ≥14 mm	6.96 (1.93–25.10)	0.003
LVEF ≤40%	3.91 (1.98–7.74)	0.0001
AL vs ATTRm	2.52 (0.91–6.95)	0.075
ATTRwt vs ATTRm	1.78 (0.66–4.77)	0.254

Multivariate Cox proportional hazards regression model for heart failure hospitalization including CAMY-HF components (low QRS voltage, interventricular septal thickness ≥14 mm, and LVEF ≤40%) and amyloid subtype (with ATTRm as reference category). Data are presented as hazard ratio (HR) with 95% confidence interval (CI). A two-sided p-value < 0.05 was considered statistically significant.

https://doi.org/10.3390/xxxxx
